# Efficacy and safety of botulinum toxin type A in distraction osteogenesis of the lower extremities: a meta-analysis of randomized controlled trials

**DOI:** 10.1186/s12891-022-05175-2

**Published:** 2022-03-25

**Authors:** Yu-Chi Su, Yao-Hong Guo, Pei-Chun Hsieh, Yu-Ching Lin

**Affiliations:** 1 National Cheng Kung University Hospital, College of Medicine, National Cheng Kung University, Tainan, Taiwan; 2 Department of Physical Medicine and Rehabilitation, National Cheng Kung University Hospital, College of Medicine, National Cheng Kung University, Tainan, Taiwan; 3 Department of Physical Medicine and Rehabilitation, College of Medicine, National Cheng Kung University, Tainan, Taiwan

**Keywords:** distraction osteogenesis, botulinum toxin, limb lengthening, deformity correction

## Abstract

**Background:**

To explore the efficacy and safety of botulinum toxin in patients who received distraction osteogenesis of the lower extremities.

**Methods:**

We searched the PubMed, Medline, Cochrane Library, and Web of Science databases for randomized controlled trials that administered botulinum toxin to individuals who underwent distraction osteogenesis of the lower limbs. The final search was conducted on July 6, 2021. Quality assessments were conducted using the Cochrane risk of bias tool and the Jadad scale. We performed random-effects meta-analysis to calculate the standardized mean differences (SMDs) and confidence intervals (CIs) of the pooled effect sizes, and subgroup analysis and meta-regression were performed for potential moderators.

**Results:**

Our analysis of four randomized controlled trials, which enrolled a total of 257 participants, revealed that the difference in pain during the distraction phase was not statistically significant between groups (SMD, − 0.165; 95% CI, − 0.379 to 0.050, *p* = 0.133, I^2^ = 0.0%). The meta-regression analyses did not find any influence on the effect size, considering age (β = − 0.0092; *p* = 0.61) and the amount of lengthening (β = 0.0023; *p* = 0.99). Subgroup analysis did not reveal difference between different doses of botulinum toxin and single or multi-site study design. An analysis of two randomized controlled trials enrolling a total of 177 individuals demonstrated a limited effect of botulinum toxin in reducing postoperative pain (SMD, − 0.239; 95% CI, − 0.641 to 0.162, *p* = 0.24, I^2^ = 37.6%), total adverse events (SMD, − 0.207; 95% CI, − 0.505 to 0.090, *p* = 0.17, I^2^ = 0.0%), and infection of pin site (SMD, − 0.131; 95% CI, − 0.428 to 0.165, *p* = 0.39, I^2^ = 0.0%). No botulinum toxin–related adverse events were reported.

**Conclusions:**

The current evidence does not support the administration of botulinum toxin in patients who receive distraction osteogenesis of the lower limbs. However, we were unable to draw decisive conclusions because of the limitations of our meta-analysis. Future well-designed, large-scale randomized controlled trials are necessary to confirm our conclusions.

**Supplementary Information:**

The online version contains supplementary material available at 10.1186/s12891-022-05175-2.

## Background

Distraction osteogenesis (DO) is a well-established treatment for many disorders and yields favorable outcomes in the majority of cases, including lengthening and deformity correction of the lower limbs [1]. The procedure typically comprises osteotomy, latency, distraction, and consolidation [2]. When applied to the lower extremities, musculoskeletal and neurovascular complications may occur [3], and the rate differs depending on the etiology of the underlying disease [4, 5]. One frequent complication is contracture of muscles and adjacent soft tissues, which may result from tension generated during lengthening and may also lead to joint luxation, axial deviation, and joint stiffness [3]. Previous studies have commonly observed loss of motion in the joints after the procedure of DO [6] although most patients of contracture gradually resolve through vigorous physiotherapy and orthosis use [7]. However, some cases require surgical intervention to improve contracture and other comorbidities, especially those with a larger amount of lengthening [8, 9]. Pain is also a concern after DO intervention, which may arise from the stretching of the periosteum, muscle spasms, contractions due to wire or pin transfixation, and inflammation of the soft tissue and bone [10]. Unlike the pain after most surgeries that decreases rapidly with time, patients who undergo DO experience a longer period of discomfort because of the long time frame of the procedure [11]. In individuals receiving DO, the discomfort caused by osteotomy makes up the pain in the first few days, and the ache during distraction phase likely originates from soft tissue elongation [10]. Severe pain may even interrupt the lengthening process [10]. Hence, identification of new methods to overcome above-mentioned problems including the contracture and pain associated with DO is warranted to improve joint stiffness and discomfort.

Botulinum neurotoxin (BoNT) blocks stimulus-induced acetylcholine release at presynaptic nerve terminals and has been demonstrated to be safe and effective for treating muscle overactivity and musculoskeletal pain [12–14]. Theoretically, BoNT may also decrease pain and complications resulting from muscle tension in patients who undergo DO of the lower extremities. Several trials have applied BoNT in this group of patients but presented conflicting results [2, 15–17], and a study hypothesized that such difference may derive from the different doses of BoNT between studies [17]. Besides, another trial mentioned that distinct postoperative pain management approaches between hospitals in a multi-site study may cause variation in the results of pain [2]. Finally, risk factors for surgical complications after DO, such as age and amount of distraction, may as well interfere the effectiveness of BoNT in DO [18]. However, no previous articles have comprehensively evaluated the safety, effectiveness and their moderators of BoNT in such patients.

We conducted a systematic review and meta-analysis that investigated the outcomes of BoNT administration in patients who underwent DO of the lower extremities.

## Methods

This systematic review was conducted according to the Preferred Reporting Items for Systematic Review and Meta-Analysis (PRISMA) guidelines [19]. The protocol was registered on the International Platform of Registered Systematic Review and Meta-analysis Protocols (INPLASY). The registration number is INPLASY2021110027.

### Eligibility criteria

Inclusion criteria were patients who underwent DO of lower extremities and received BoNT administration. The control group should be treated with placebo. The studies should at least have pain as one of the outcomes. Randomized controlled trials were eligible for inclusion.

We excluded studies without mentioning the surgical indications of DO and the distention methods used during the trials. Besides, studies not published in English were also excluded.

### Search strategy

PubMed, Medline, Cochrane Central Register of Controlled Trials, and Web of Science were searched with the language restricted to English. The key terms were “distraction osteogenesis” AND “botulinum toxin.” The search time was from database inception to the search date. The final search was conducted on July 6, 2021 (see Supplementary file 1 for the full search strategy).

### Study selection and data extraction

The first three authors (YCS, YHG and PCH) reviewed the titles and abstracts of applicable studies independently. The senior author (YCL) made the final decision if a consensus could not be reached through discussion. The following data were extracted from the selected studies using a data collection sheet: the first author, year of publication, demographic information, classification of deformity, surgical location and type, amount/rate of lengthening, dosage of BoNT, dilution method of BoNT, commercial form of BoNT, site of injection, comparative regimen, pain, range of motion (ROM) of joints, adverse events, and other clinical assessments and radiological outcomes. We contacted the authors of related articles if necessary to resolve any uncertainties.

### Quality assessment

We assessed the selected studies using the Cochrane risk of bias tool [20] and the Jadad scale [21] for randomized controlled trials. Disagreements between results were resolved by discussion. The senior author (YCL) determined the results if a consensus was not reached. Reviewer Manager version 5.3 was used to visualize the risk of bias in a graph and summary table.

### Statistical analysis

All articles reporting the outcomes in interest were included in quantitative synthesis. For researches missing standard deviation and mean values, we made use of available *p*-value and sample size information [22]. The primary outcome was a reduction in pain during the distraction phase after injection, represented as standardized mean differences (SMDs) and 95% confidence intervals (CIs). The secondary outcomes were the rate of total adverse events, infection of pin site, and maximal pain on postoperative day 1 after DO; these outcomes are also represented as SMDs and 95% CIs. The effect sizes were pooled using a random-effects model. In addition, we conducted a random-effects meta-regression to explore whether the primary outcome varied depending on different study characteristics. These characteristics comprised continuous variables including age and amount of lengthening. As for the categorical variables such as the dose of BoNT and single or multi-site study design, the included trials would be grouped first, and the summarized effect sizes of the subgroups would be calculated separately. Nonoverlapping 95% CIs indicated significant difference between subgroups. Studies would be defined as multi-site research if the surgeries and distraction procedures in the trials were conducted in more than one hospital, and studies with surgeries and distraction procedures done in only one hospital would be defined as single-site research. The I^2^ statistic was used to assess between-study heterogeneity, which was defined as low, moderate, or high using cutoff values of 50 and 75% [23]. We used funnel plots and Egger’s test to assess publication bias [24], and a two-tailed *p* value lower than 0.1 was regarded as statistically significant [25]. A sensitivity analysis was performed for the primary outcome by removing one trial at a time and analyzing the remaining trials to estimate whether the effect resulted from a single study. We used Comprehensive Meta-Analysis Software version 3 (Biostat, Englewood, NJ, USA) for all analyses.

### Certainty of evidence

The certainty of the evidence of the primary outcome was assessed by the Grading of Recommendations Assessment, Development and Evaluation (GRADE) methodology. The results begin as high certainty, because our study included only randomized controlled trials. The final rating depends on the overall risk of bias, imprecision, inconsistency, indirectness, and publication bias [26].

## Results

### Study selection and description

**Fig. 1:**
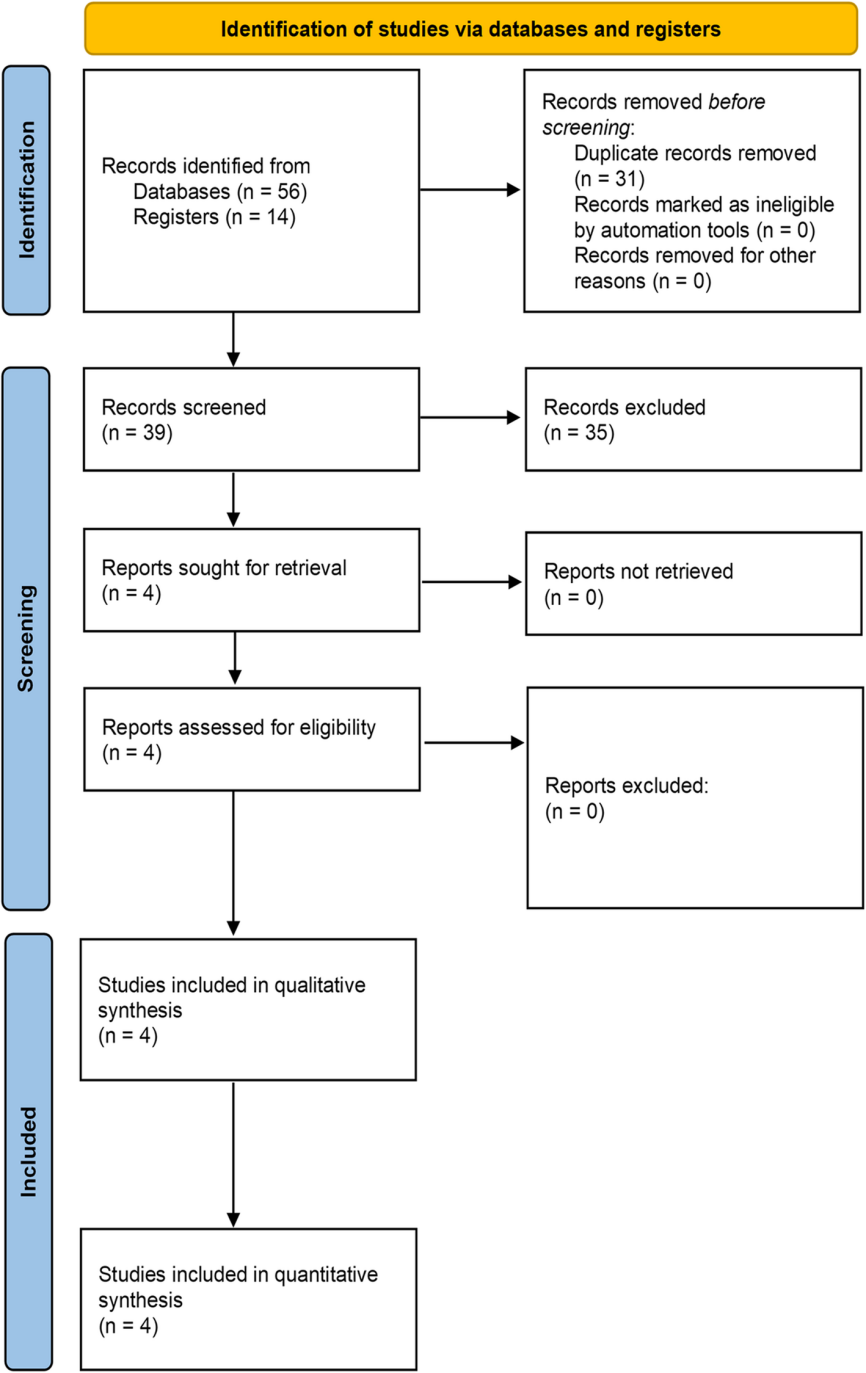
Literature screening process and results

We identified 70 articles in the initial search, four of which met our inclusion criteria (Fig. 1). The level of evidence was assigned according to the Oxford Centre for Evidence-Based Medicine 2011, and all four studies [2, 15–17] were rated as level 2. The summarized information of trials included in our systematic review is shown in Table 1.

**Table 1:** Characteristics of all included researches

Research	Country	Study design	Age at the time of surgery (years)	Classification of deformity	Gender (Male/Female)	Type of surgery	Location of surgery	Amount of lengthening (cm)	Rate of lengthening (mm/day)	Lengthening technique	Last follow-up time
Hamdy et al., 2009 [2]	Canada and United states	Randomized, double blinded, controlled trial	BoNT: 12.1 (4.5), control: 14 (3.4)	Congenital/acquired deformity; BoNT: 25/6, control: 18/3	BoNT: 20/11, control: 17/4	Lengthening / Angular correction and/or lengthening / Clubfoot correction; BoNT: 17/8/6, control: 8/10/3	Femur/tibia /foot; BoNT: 6/19/6, control: 8/9/4	BoNT: 5.9 (2.3), control: 4.1 (1.9)	NR	distraction with a fixator (either circular or uniplanar)	3 months post-frame removal
Lee et al., 2014 [15]	Republic of Korea	Randomized, double blinded, controlled trial	23 (range: 16–35)	NR	31/5	Bilateral tibial lengthening for familial short stature	Tibia	BoNT: 6.4 (range: 4.5–8.1), control: 6.4 (range: 4.3–8.1)	BoNT: 0.69 (range: 0.5–0.96), control: 0.68 (range: 0.51–0.97)	LATN or LON	Mean: 30 months (range: 24–39)
Hamdy et al., 2016 [16]	Canada and United states	Randomized, double blinded, controlled trial	BoNT: 11.8 (3.3), control: 13.3 (4.0)	Congenital/acquired deformity; BoNT: 47/12, control: 44/18	BoNT: 30/32, control: 42/21	Lengthening / deformity correction / deformity correction and lengthening; BoNT: 28/10/24, control: 38/5/20	Femur / tibia / foot / tibia and foot; BoNT: 18/35/7/2, control: 29/30/2/2	BoNT: 4 (1.9), control: 4.4 (1.6)	NR	distraction with a fixator (either circular or uniplanar)	3 months post-frame removal
Park et al., 2016 [17]	Republic of Korea	Randomized, double blinded, controlled trial	26 (8)	NR	35/9	Bilateral femoral lengthening for familial short stature	Femur	BoNT: 5.4 (0.6), control: 5.5 (0.6)	BoNT: 1.28 (0.14), control: 1.27 (0.12)	Intramedullary lengthening nails	Mean: 26 months (8, range: 14–40)

Results are given as mean (SD), unless otherwise noted; *BoNT* Botulinum neurotoxin group, *LATN* Lengthening and then nailing, *LON* Lengthening over nail, *NR* Not reported, *SD* Standard deviation

In all four studies, BoNT and normal saline was administered once intraoperatively in intervention and control group respectively. Hamdy et al. [2] recruited 52 patients with lower limb deformities in a multi-site clinical trial. The surgical locations were the femur, tibia, and foot. The quadratus femoris and medial hamstrings were injected for femoral lengthening, and the gastrocnemius and soleus were injected for tibial lengthening and clubfoot correction. This trial did not mention the brand name of BoNT, but the context and dosage suggested that it was likely onabotulinumtoxinA [16, 27]. The dosage was 10 units per kilogram of body weight with an upper limit of 400 units. Results showed a trend of pain reduction at mid-distraction, less parenteral pain medication use, improved quality of life, higher functional mobility scores, and fewer major adverse events in patients who received BoNT compared with controls, although these differences were not statistically significant. Because of these results, Hamdy et al. [16] conducted another larger double-blind, multicenter, randomized controlled trial of 125 children. The surgical locations, dose of onabotulinumtoxinA, and injected muscles were similar to their former research. Compared with placebo, patients who received BoNT experienced lower maximum pain on the first postoperative day, and a lower rate of pin site infection was observed in the tibial lengthening group. However, quality of life, mid-distraction pain, functional mobility scores, bone healing index, ROM of adjacent joints, and rate of total adverse events did not differ significantly between groups.

Lee et al. [15] conducted a single-site study of 36 participants with familial short stature who underwent bilateral tibial lengthening by the same surgeon. A 200-unit dose of onabotulinumtoxinA was administered to the gastrocnemius and soleus. The results did not reveal any differences in pain, calf circumference, or ROM of the knee and ankle. Park et al. [17] conducted a single-site study of 44 patients who received bilateral femoral lengthening for familial short stature by a single surgeon. A 200-unit dose of onabotulinumtoxinA was injected into the quadratus femoris. No differences in pain, thigh circumference, or ROM of the hip and knee were reported (Table 2).

**Table 2:** Summary of extracted data from randomized controlled trials

Study	Time of BoNT injection	Commercial forms	Dilution method	Injection dose (U)	Injection sites and methods	Outcome measurement
Hamdy et al., 2009 [2]	Once, intraoperatively, before extubation	BoNT: NR (type A), control: normal saline	100 U/ml with normal saline	10 U per kilogram body weight, up to 400 U (200 U per muscle)	Femoral lengthening: quadriceps, medial hamstrings Tibial lengthening/correction, clubfoot correction: gastrocnemius, soleus	Postoperative pain and analgesics in the first four days; pain, QoL, Functional Mobility Scores at baseline, mid-distraction, mid consolidation, frame removal and three months post-frame removal; total adverse events, BoNT related adverse events, pin-site infection
Lee et al., 2014 [15]	Once, intraoperatively, at the end of all surgical procedures	OnabotulinumtoxinA, control: normal saline	10 U/ml with normal saline	200 units	Six different spots evenly at the gastrocnemius and soleus, performed manually without instruments	Pain, calf circumference, range of motion of knee and ankle joint every 2 weeks during the distraction phase and every month thereafter; BoNT related adverse events
Hamdy et al., 2016 [16]	Once, intraoperatively, before extubation	OnabotulinumtoxinA, control: normal saline	100 U/ml with normal saline	10 U per kilogram body weight, up to 400 U (200 U per muscle)	Femoral lengthening: quadriceps, medial hamstrings Tibial lengthening/correction, clubfoot correction: gastrocnemius, soleus	Postoperative pain and analgesics in the first four days; pain, QoL, Functional Mobility Scores, muscle strength, range of motion of knee hip and ankle at baseline, mid-distraction, mid consolidation, frame removal and three months post-frame removal; total adverse events, BoNT related adverse events, pin-site infection
Park et al., 2016 [17]	Once, intraoperatively, at the end of all surgical procedures	OnabotulinumtoxinA, control: normal saline	10 U/ml with normal saline	200 units	Seven different spots evenly on the anterior thigh in the quadriceps muscle, performed manually without instruments	Pain, thigh circumference, and range of motion of knee and hip joint once per week during the distraction phase and every month thereafter; BoNT related adverse events

*BoNT* Botulinum neurotoxin, *NR* Not reported, *QoL* Quality of life, *U* Units

### Risk of bias assessment

**Fig. 2:**
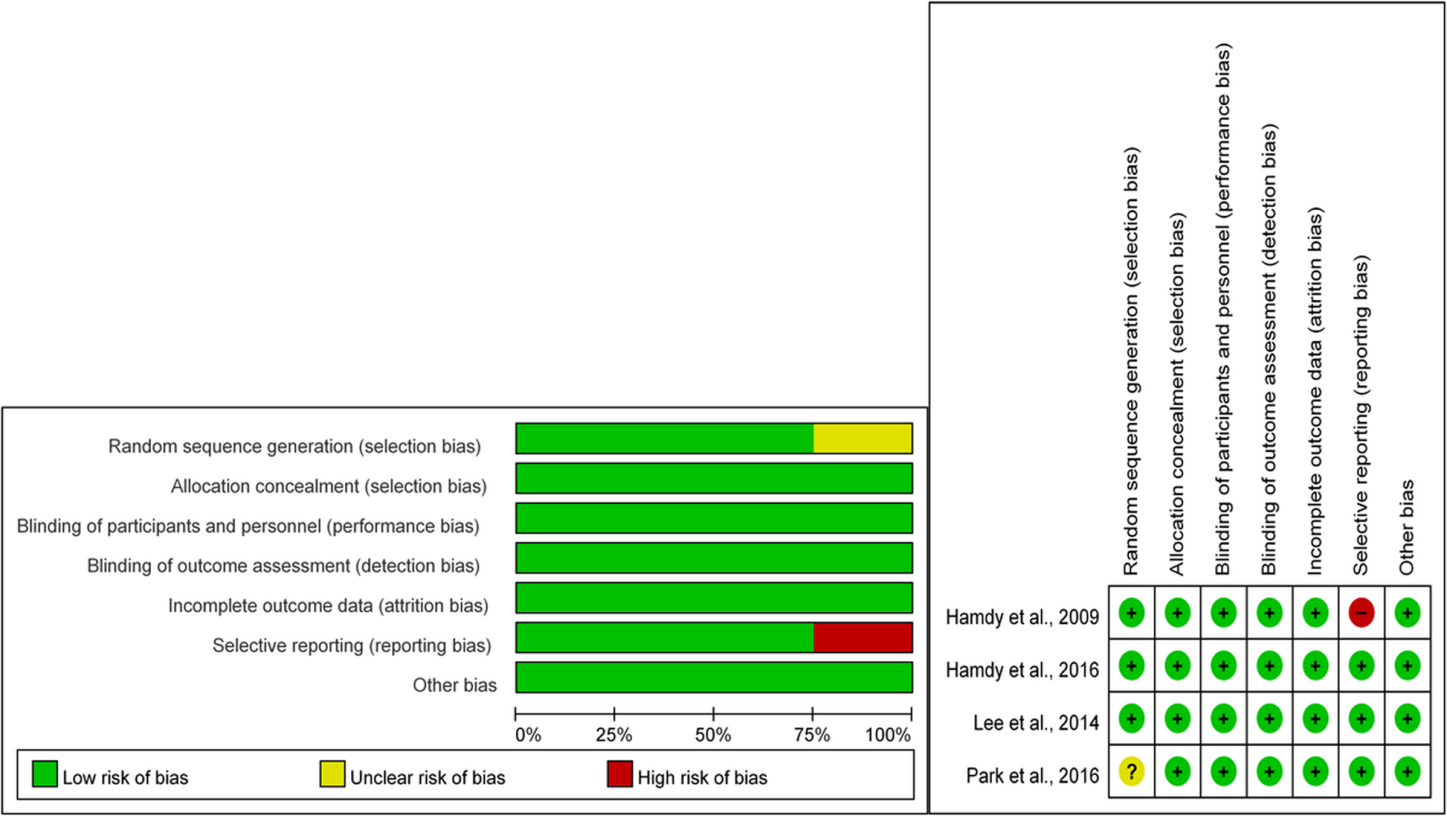
Summary graph and table for risk of bias

Among the four randomized controlled trials, one [17] revealed unclear random sequence generation. Another study [2] demonstrated a high risk of selective reporting for not mentioning the results of ROM. All studies were classified as low risk for allocation concealment, incomplete outcome data, blinding of participants, and outcome assessment (Fig. 2). As for the Jadad scale, three trials scored 5, while Park et al. scored 4 because they did not mention about the details of sequence generation in their manuscript [17] (Table 3).

**Table 3:** Risk of bias assessed by Jadad scale

	Hamdy et al., 2009 [2]	Lee et al., 2014 [15]	Hamdy et al., 2016 [16]	Park et al., 2016 [17]
Described as randomized^a^	1	1	1	1
Described as double blinded^a^	1	1	1	1
Description of withdrawals^a^	1	1	1	1
Randomization method described and appropriate^b^	1	1	1	0
Double-blinding method described and appropriate^b^	1	1	1	1
**Score**	**5**	**5**	**5**	**4**

^a^A study scores 1 for “yes” and 0 for “no”

^b^A study scores 0 if no description is given, 1 if the method is both described and appropriate, and − 1 if the method is described but inappropriate

### Data extraction: qualitative synthesis

None of the four trials demonstrated that BoNT was effective in reducing pain in the distraction phase. Among the three studies reporting ROM, none reported a difference between the placebo and BoNT. Two articles analyzed postoperative pain, analgesic medication after surgery, quality of life, functional mobility scores, total adverse events, and the rate of pin site infection. One [16] research reported a reduction in infection of pin site in the tibial lengthening group and maximum pain on the first day after surgery, whereas neither study reported significant differences in the other outcomes.

None of the four studies reported BoNT-related adverse events. Two trials revealed no difference in lower limb circumference between the intervention and control groups.

### Data extraction: quantitative synthesis

The meta-analysis for the primary outcome comprised four randomized controlled trials [2, 15–17]. Meta-regression analyses were conducted using mean age and amount of lengthening. Subgroup analysis included dose of BoNT and single or multi-site study design. The number of participants was 257, and the mean age ranged from 12.6 to 26 years old. The amount of lengthening ranged from 4.2 to 6.4 cm. The dosage of BoNT was 200 units per participant in two studies and 10 units/kg of body weight with an upper limit of 400 units in the other two researches. The amount of lengthening ranged from 4.2 to 6.4 cm. Two randomized controlled trials [2, 16] with 177 participants were included in the meta-analysis of secondary outcomes. The average age ranged from 12.6 to 12.9 years (Table 1).

**Fig. 3:**
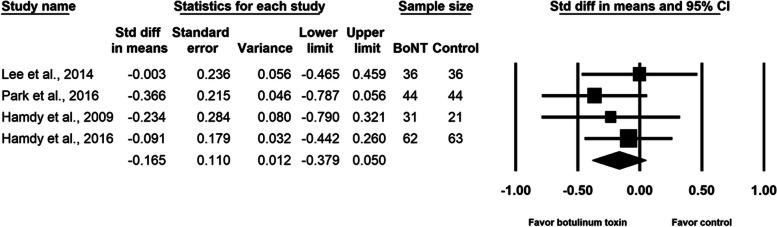
Forest plot of standardized mean differences in reduction of pain during distraction phase

**Fig. 4:**
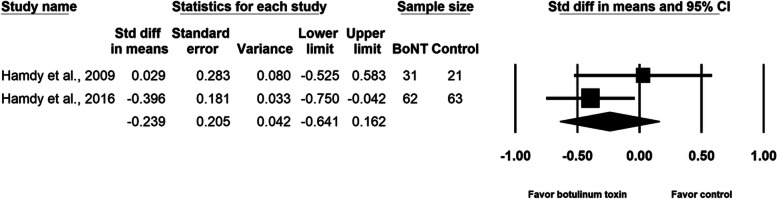
Forest plot of standardized mean differences in reduction of maximal pain on post-operative day one

**Fig. 5:**
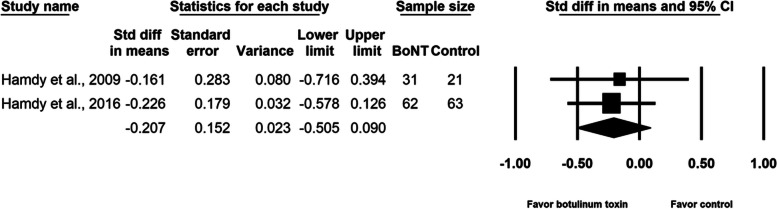
Forest plot of standardized mean differences in reduction of total adverse events per patient

**Fig. 6:**
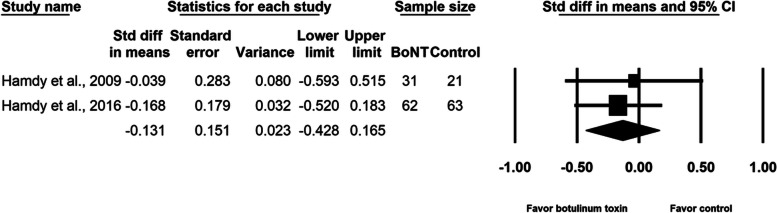
Forest plot of standardized mean differences in reduction of pin-site infection per patient

**Fig. 7:**
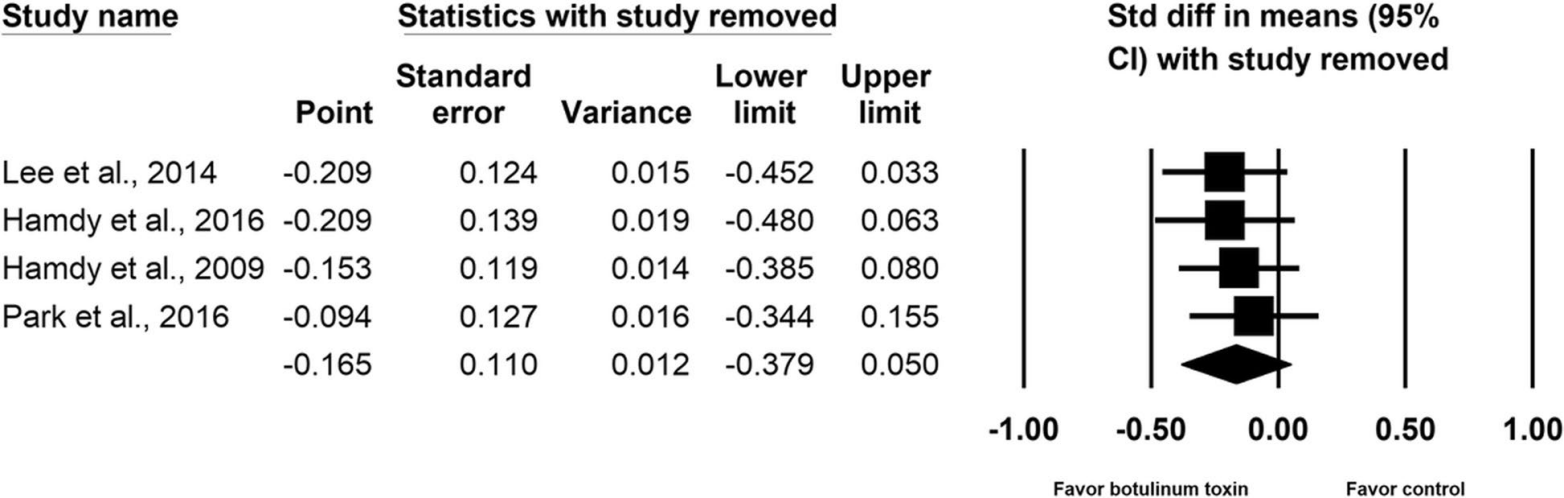
Sensitivity analysis by one-study-removed analysis in reduction of pain during distraction phase

BoNT injection did not relieve pain during the distraction phase (SMD, − 0.165; 95% CI, − 0.379 to 0.050, *p* = 0.13, I^2^ = 0.0%; Fig. 3). The meta-regression analyses did not reveal any influence on effect size considering age (β = − 0.0092; *p* = 0.61) and amount of lengthening (β = 0.0023; *p* = 0.99). As for the subgroup analysis for different doses of BoNT, the effect sizes were − 0.132 (95% CI, − 0.429 to 0.165) for the two studies [2, 16] using 10 units/kg of body weight and − 0.197 (95% CI, − 0.552 to 0.157) for the two studies [15, 17] using 200 units per participant. Two studies [2, 16] were multi-site research and the other two [15, 17] were single-site research. Summarized effect sizes of the two subgroups were − 0.132 (95% CI, − 0.429 to 0.165) and − 0.197 (95% CI, − 0.552 to 0.157) respectively. No significant difference between groups was observed in maximal pain on postoperative day 1 (SMD, − 0.239; 95% CI, − 0.641 to 0.162, *p* = 0.24, I^2^ = 37.6%; Fig. 4). Neither the total number of adverse events (SMD, − 0.207; 95% CI, − 0.505 to 0.090, *p* = 0.17, I^2^ = 0.0%; Fig. 5) nor the pin site infection rate (SMD, − 0.131; 95% CI, − 0.428 to 0.165, *p* = 0.39, I^2^ = 0.0%; Fig. 6) differed between groups after BoNT administration. The primary and secondary outcomes demonstrated low between-study heterogeneity. No publication bias was detected for the primary outcome by Funnel plot and Egger’s test (*p* = 0.8, Supplementary file 1), whereas the publication bias for secondary outcomes could be not assessed because of the small number of trials. In the sensitivity analysis for the primary outcome, the SMD ranged from − 0.209 (95% CI, − 0.452 to 0.033, *p* = 0.09; Fig. 7) when the study by Lee et al. [15] was excluded, to − 0.094 (95% CI, − 0.344 to 0.155, *p* = 0.46) when the trial by Park et al. [17] was removed.

### Certainty of evidence

The certainty of the evidence of the decrease of pain during the distraction phase by BoNT revealed a moderate quality of evidence. The level was downgraded because of the large 95% CI (Table 4).

**Table 4:**
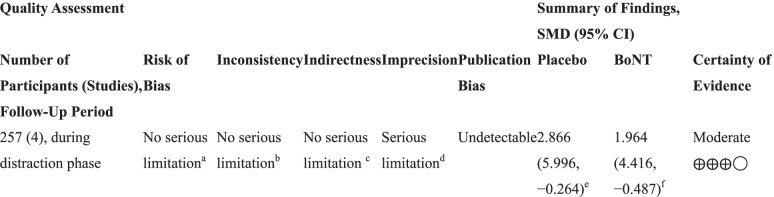
Certainty of evidence for decrease of pain during distraction phase

*BoNT* Botulinum neurotoxin, *CI* Confidence interval, *SMD* Standardized mean difference

^a^Most studies included scored low risk of bias during assessment

^b^I^2^ score was below 50%

^c^No indirectness was detected in this outcome

^d^The upper and lower limit of 95% CI ranged from favoring placebo to favoring BoNT

^e^This was calculated by pooling the placebo group of the 2 articles included in the primary outcome presenting sufficient data (Hamdy et al., 2009, 2016), comparing the pain score before surgery and during distraction phase

^f^This was calculated by pooling the BoNT group of the 2 articles included in the primary outcome presenting sufficient data (Hamdy et al., 2009, 2016), comparing the pain score before surgery and during distraction phase

## Discussion

In our systematic review and meta-analysis, BoNT did not relieve pain during the distraction phase, and age, amount of lengthening, and dose of BoNT were not correlated with significantly different effect sizes. The sensitivity analysis further indicated the stability of the results. Moreover, pain on postoperative day 1, total adverse events, and pin site infection rate did not differ after BoNT administration. However, our results cannot support or discourage the use of BoNT in patients who receive DO of lower extremities because of insufficient data from limited researches in the literature.

Pain frequently occurs during the distraction phase in limb lengthening [10]. The sources of such pain are still unclear, may be related to tension in soft tissues and excessive contraction of muscles [10]. BoNT is effective in muscle pain associated with muscle overactivity [28, 29]. Therefore, the non-significant pooled effect size in our meta-analysis may help exclude the role of excessive muscle contraction in pain during bone distraction. Previous studies have also demonstrated the absence of increased muscular activity throughout distraction by electromyography [30, 31]. These findings may imply that increased tension during bone lengthening stemmed from the relative shortening of muscles and tendons in relation to the elongation of bones, as compared to muscle overactivity, contributes more to the discomfort of patients after DO surgery [10].

Although the summarized effect of decrease in pain on the first day after surgery was nonsignificant, we believe that the evidence so far was still insufficient to rule out the potential analgesic effect of BoNT. Several uncontrolled factors, such as the techniques of the orthopedic surgeons and the pain control regimens, may influence the final results of pain alleviation [2]. However, we could not prove this by subgroup analysis due to the small number of studies available. Further trials with more detailed information and standardized protocol of pain control and other items are needed to clarify the analgesic effect of BoNT on the first few days after DO.

Although current evidence do not support the use of BoNT in DO in the aspect of ROM, we believe that BoNT might still have the potential to play a role in joint contracture after DO of the lower limbs. Previous researches had reported that patients often experienced joint stiffness during the distraction phase, which gradually resolved after the individuals entered the consolidation phase with the help of intensive physiotherapy and splinting throughout the entire process [6, 7, 10]. However, articles have found that patients with excessive lengthening of the tibia and femur were more prone to sustained joint stiffness despite splinting and physical therapy, which requires further surgical correction [8, 9]. The pathophysiology of the formation of joint stiffness recalcitrant to conservative treatments is still unclear. No direct evidence has been published to date that excludes the role of muscle overactivity, which can be treated with BoNT [29]. Interestingly, in patients with hip and knee contractures following arthroplasty who were refractory to physical therapy, BoNT improved ROM [32, 33]. Future research is necessary to delineate the role of BoNT in decreasing the risk of or relieving sustained joint stiffness.

As for the safety of BoNT in DO, our results may reassure future studies to administer BoNT to patients treated with DO. Interestingly, one study even revealed fewer pin site infections in the tibial lengthening subgroup after BoNT administration [16]. In children with tibial circular external fixators, the pin site infection rate was higher in the periarticular region compared with that in the diaphysis, possibly because of increased soft tissue motion around the joints [34]. BoNT might achieve its effect by muscle immobilization to decrease joint movements. However, more research are warranted to prove this hypothesis.

Our meta-analysis has several limitations. First, the statistical power was limited by the small sample size of four included articles. However, all four were randomized controlled trials with reliable levels of evidence. Second, two studies [2, 16] reported outcomes in different phases of DO, and the other two [15, 17] reported results by the number of weeks after BoNT injection. So further analyses of outcomes during the various phases were impossible. Third, the underlying deformities of the participants, which have been related to surgical outcomes and total complications, differed between the studies [5, 10]. Fourth, distinct surgical devices and lengthening technical options varied within and between trials. These differences were found to be linked to pain, joint stiffness, bone healing, and complications such as infection [10]. However, we could not analyze these factors due to the high heterogeneity between studies and relatively small numbers of trials. Fifth, limb lengthening is a complicated procedure that requires a high level of operator experience [10]. This presents difficulties for standardization between individual surgeons and hospital [35], which was the case in two multi-center studies in our review [2, 16]. To overcome these obstacles, future research should address surgical learning and clustering effects to refine the calculation of the treatment effect of BoNT [36]. Finally, no BoNT-related adverse events were reported in the studies included in our review. However, the number of patients may still be insufficient to detect rare and severe adverse events [37].

The implication, particularly clinical impact of our study is to provide evidence to oppose the routine use of BoNT in patients undergoing DO. However, we could not exclude the possibility that BoNT might be effective in certain subgroup of patients. Hence, we provided some recommendations for clinical practice and future studies to overcome the limitations noticed in our review. First, homogenous participants, standardized pain control methods, and identical surgical techniques conducted by surgeons with similar experience in DO are necessary. Second, the effectiveness of BoNT in patients with high risk of joint contracture or even in those with physiotherapeutic recalcitrant contracture needs further research. Finally, the application of BoNT in general population receiving DO of the lower limbs should not be encouraged in clinical settings before more evidence emerges in the future.

In individuals receiving DO of the lower extremities, no significant effects of BoNT type A on pain, total adverse events, and pin site infections after surgery were observed. However, our results cannot support or discourage the use of BoNT in these patients because of the limitations of our meta-analysis. Future well-designed, large-scale randomized controlled trials are necessary to confirm the role of BoNT in surgery with distraction osteogenesis of the lower extremities.

## Supplementary Information

**Additional file 1.**

## Data Availability

Not applicable. All data used for analysis was derived from openly published articles listed in our manuscript.

## Abbreviations

BoNT: Botulinum neurotoxin
CI: Confidence interval
DO: Distraction osteogenesis
ROM: Range of motion
SMD: Standardized mean differences
